# Psychiatric disorders during premature ovarian failure and the benefits of exerkines

**DOI:** 10.1192/j.eurpsy.2025.1567

**Published:** 2025-08-26

**Authors:** N. Bouayed Abdelmoula

**Affiliations:** 1 Genomics of Signalopathies at the Service of Precision Medicine, LR23ES07, Medical University of Sfax, Sfax, Tunisia

## Abstract

**Introduction:**

Premature ovarian failure (POF) is manifested by menstrual troubles and menopause symptoms including psychiatric burdens such as depression, anxiety, and decreased perceived psychosocial support. POF predisposes to many health risks, including decreased bone density and early progression of cardiovascular disease. Hormono-therapy of replacement of ovarian sex steroids is indicated until the normal age of natural menopause.

**Objectives:**

The aim of our work was to study the benefits of increased physical activity and regular exercise in POF women and to show how exercise produces positive biological effects on their psychiatric health through the contribution of the exerkines network.

**Methods:**

We conducted a comprehensive review of the scientific literature with the following keywords: Premature ovarian failure (POF), exerkines and psychiatric health.

**Results:**

Increased physical activity and regular exercise are accompanied by the discharge of exerkines that represent an intricate network of signaling molecules or kines released into the systemic circulation during and after exercises. These molecules derived from various tissues (myokines, batokines, etc…) and organs (organokines) such as skeletal muscle, white and brown fats, liver, bones, nervous system, heart, etc…) act in different ways on the body: autocrine, paracrine or endocrine manners. Their roles in metabolic regulation, neuroprotection, and muscle adaptation highlight the importance of physical activity in maintaining health and preventing disease. Generally, it is shown that exercise can positively affect hormonal balance, homeostasis and overall health. It is also reported that physical activities promote stress reduction and hormonal regulation. Consistency and moderation are key to reaping the benefits of exercise for ovarian health. We found no human studies reporting the action of exerkines and myokines in women with POF. Some studies reporting the effects of physical exercise without further details on exerkines were found in women during and before menopause. The relationship between ovarian function and exercise, as well as exerkines levels and proteomics, was non-existent in humans and rare in animal models. Other research indicates that exercise has a positive impact on mental health, improving mood, self-esteem, and reducing stress and anxiety levels, through the improvement of the production of endorphins and neurotransmitters, the mitochondrial function and the hypophyso-hypothalamo–adrenal axis regulation interaction response to stress.

**Conclusions:**

We conclude that human scientific studies in women with ovarian deficiency should now be carried out to investigate the types of exercise to be recommended in these women to improve their mental health. These studies should benefit from next-generation technologies such as proteomics and interactomics.

**Disclosure of Interest:**

None Declared

